# The World Health Organization and the global standardization of medical training, a history

**DOI:** 10.1186/s12992-021-00733-0

**Published:** 2021-08-28

**Authors:** George Weisz, Beata Nannestad

**Affiliations:** Cotton-Hannah Chair in the History of Medicine, Department of Social Studies of Medicine, McGill University, 3647 Peel Street, Montreal, Quebec Canada

**Keywords:** World Health Organization, World Federation for Medical Education, Medical training, Standardization, Accreditation, Colonialism

## Abstract

**Background:**

This article presents a history of efforts by the World Health Organization and its most important ally, the World Federation for Medical Education, to strengthen and standardize international medical education. This aspect of WHO activity has been largely ignored in recent historical and sociological work on that organization and on global health generally.

**Methods:**

Historical textual analysis is applied to the digitalized archives and publications of the World Health Organization and the World Federation for Medical Education, as well as to publications in the periodic literature commenting on the standardization of international medical training and the problems associated with it.

**Results:**

Efforts to reform medical training occurred during three distinct chronological periods: the 1950s and 1960s characterized by efforts to disseminate western scientific norms; the 1970s and 1980s dominated by efforts to align medical training with the WHO’s Primary Healthcare Policy; and from the late 1980s to the present, the campaign to impose global standards and institutional accreditation on medical schools worldwide. A growing number of publications in the periodic literature comment on the standardization of international medical training and the problems associated with it, notably the difficulty of reconciling global standards with local needs and of demonstrating the effects of curricular change.

## Background

Globalization takes many forms. One that has been largely ignored by historians and social scientists has centered on the international standardization of medical training. This is a complex phenomenon encompassing a variety of practices. Some like bi-lateral national agreements, partnerships among medical schools, creation of new medical schools (often for profit) appealing to an international clientele, and the setting up of local branches of elite medical schools – are fragmented, individualized and difficult to examine collectively [1]. An exception is the long-standing work of the World Health Organization (WHO) to reform medical education worldwide, which has generated a large volume of accessible sources and provides a focused perspective over 70 years.. Surprisingly, this aspect of WHO activity has been largely ignored in recent historical and sociological work on that organization [2] and on global health (GH) generally [3]. Nor is it better represented in the GH literature which tends to ignore the subject.

This is unfortunate since this history has much to tell us about the evolution of medical training, about international and global health, and about the relationship between the Global North and Global South. We propose in this article to fill this gap by examining the efforts since 1950 by the WHO and its most important ally, the World Federation for Medical Education (WFME), to strengthen and standardize international training for physicians. The work of the WHO and its allied organizations provides a unique perspective on the bumpy, crooked, and uncompleted road toward global healthcare improvement, as well as on evolving ideas and norms within both medical schools and GH institutions. It makes visible the changing strategies, persistent tensions, and competing imperatives of standardization efforts and of the international medical enterprise more generally. If it does nothing else, this paper seeks to move the question of medical training to a more central and visible place in the GH literature that is more congruent with its potential significance for the improvement of health worldwide.

## Methods

This is a work of history. That means that the methodology used is rather different than readers of the journal are used to. Rather than engaging in a systematic search of one or more databases and then choosing a number of relevant documents to report on, we as historians have collected sources using multiple techniques: targeted searches of multiple databases, of citations and bibliography in relevant publications, articles discovered accidentaly during searches on other subjects, suggestions in websites like ResearchGate or sent by colleagues, and hunches that some seemingly irrelevant sources may in fact contain useful material. This process continues even as writing and revisions proceed. The core of this article relies on the digitalized archives and publications of the WHO and the WFME available on their online web pages. The WHO IRIS database is particularly rich in documentation located by using a variety of search terms (medical training, medical schools, curriculum, and so on). These sources are not so much analyzed as *mined* for information, arguments and themes in order to *reconstruct* the history of efforts to transform training at the international level. The major sources used are cited in the notes and can be accessed to verify that our interpretation is accurate. Multiple searches over several year make us confident that few relevant sources in this digital archive have been missed. The historical narrative constitutes the “results” of the exercise.

The article goes on to discuss the small but growing number of publications in the periodic literature commenting on efforts to standardize international medical training and their associated problems, inconsistencies, and conflicting imperatives. These sources have been located through searches in multiple databases using multiple search terms (medical schools AND standardization; medical training AND guidelines; medical schools AND reform, and so on). These result in large numbers of hits but only a few that turn out to be relevant to our subject. These are supplemented by examination of the works they cite and the works that cite them, as well as undertaking fishing expeditions in influential journals like Academic Medicine and Medical Teacher. This corpus is cited in the endnotes and does not claim to be exhaustive. The critiques and arguments they make are meant to suggest the range of views that this movement has inspired.

Although WHO programs for education reform were aimed at a variety of healthcare personnel (particularly nurses and midwives, but also auxiliaries), our focus in this paper will be on the training of physicians for several reasons. First, physicians have been considered potential leaders of wider health system reform and have thus received special attention. Second efforts to reform physician training have provided models for the education of other health professionals. Third and more practically, including these occupations would make an already lengthy paper far longer. We intend to deal with these other occupations in a future article which examines the wider strategy now called Human Resources for Health and which also deals with non-training issues like labour markets and work conditions.

## Results

As we will document in the following sections, the efforts of the WHO to improve medical training internationally took place over three distinct periods. The early decades 1949–1973 were characterized by several perceived imperatives: to expand the number of health personnel, particularly in Low and Middle Income Countries (LMIC); to introduce compatibility of diplomas to facilitate the international movement of health workers; to transfer the scientific instruction characteristic of medical schools in high-income countries to lower-income nations; and to train health personnel in the tenets of public health and prevention.

A second period from 1973 to the mid 1980s associated these traditional concerns with the need to adapt medical training to the WHO’s new orientation expressed as Primary Health Care (PHC) or Health for All (HFA) and, more generally, to adapt training more closely to the needs of local healthcare systems as opposed to the native values of medical academics. It coincided as well with a new emphasis within the WHO on strengthening health systems.

A third period from the late 1980s to the present has been characterized by the attempt to operationalize these concerns and improve the quality and safety of medical practice, first through formal training standards expressed in published guidelines and then through accreditation procedures for training institutions. These were carried out in collaboration with international professional organizations, particularly the WFME, which worked closely with the regional and national units of the WHO.

New goals did not replace older ones but were added by accretion. This led to regular reiteration of many themes during all three periods. New themes did appear however and older ones were sometimes reformulated. Similarly some tensions, indeed competing imperatives, emerged early on: between evolving GH norms (focused on adapting training to local health-system needs) and the international culture of medical schools (based on what appear to be universal principles); between desire for global guidelines and insistence that education reform reflect local concerns; between the desire to provide physicians with the highest quality training, often in wealthy nations, and the wish to keep them from remaining in these countries and robbing their nations of origin of needed personnel. Other tensions have emerged more recently: notably between concern to promote self-improvement among medical schools and the quest for a regulatory framework to manage an increasingly global market for health professionals.

### The early decades

From the WHO’s beginnings, medical training was a major focus of activity. It was considered a key component of its overall ambition to improve healthcare worldwide because the chief barrier to adequate healthcare in many countries appeared to be lack of skilled personnel. The solution generally proposed was to expand and improve training facilities in order to increase the number of skilled personnel [4]. During the 1950s and 1960s, an Expert Committee on Professional Education functioned regularly, becoming in the 1970s the Division of Health Manpower [5]. The quest for greater quantity and better quality of physicians initially centered on establishing new medical schools and improving older ones. There were 533 medical schools in WHO Member States around 1950 and 717 by 1966, with the bulk of increases in Latin America and Asia. This multiplication of schools was problematical due to the absence of international standards and the consequent lack of equivalence of degrees, at a time when medical personnel were becoming internationally mobile [6].

These efforts were marked by tensions between global pressure for standardized change (modeled on developments in wealthy countries) and local needs and resources of less wealthy countries. Such tensions could be handled in various ways. One could claim that certain ideas, notably the need for training in basic medical science and preventive care, had universal applicability [7]. It was conceded that training for physicians in “developing” countries should differ from those in high-income countries, providing a wider skillset to treat and prevent diseases and provide leadership for teams of auxiliaries [8]. Nonetheless, a series of expert committees and conferences sought during these decades to develop and transmit principles that could be applied worldwide [9]. WHO co-sponsored conferences organized by the World Medical Association, while organizing its own meetings on medical education, including regional conferences in Teheran (1962), Manila (1964), and Yaounde (1966) [10] and an inter-regional conference in Geneva (1964) [11]. Among curriculum issues discussed at such meetings were the length of studies, the pre-medical curriculum, and the introduction of clinical, laboratory and bedside work to supplement lectures and reduce reliance on memorization [12].

Application of global principles was fostered by a variety of programs. One involved sending foreign personnel abroad. WHO sent professors to needy institutions as part of multi-disciplinary teams (for shorter periods) or as individuals (usually for longer periods). Their mandates were typically to train the teachers who could replace them. WHO visiting professors reorganized departments, taught courses, and initiated research projects, all with the goal of training local staff to take over. In addition, WHO provided teaching equipment and medical literature [13]. From 1950 to 1956, WHO organized or co-organized 129 courses with some 2500 participants [14]. The University of Copenhagen became a center for the teaching of anesthesiology, while a centre for training in biostatistics was set up in Santiago, Chile. A two-year training course for teachers of preventive medicine was organized at Harvard for personnel of the Southeast Asia region [15].

An extensive program of fellowships was established at WHO which financed, among other activities, participation in training programs, educational meetings and conferences (see below). Fellowships were seen as critical to the improvement of healthcare training. Fellows brought back new ideas and techniques,

which they pass on to others in their countries. They introduce new methods in existing services and make them more effective. They establish health services new to their countries, and they undertake research. Perhaps most important, a very high percentage of fellows are taking an active part in training programmes and thus passing on to others the benefits they have themselves received.” [15]

To ensure the success of these fellowship, applications from individuals had to be sponsored by their governments, which were required to provide a formal declaration that training would be put to practical use once fellows returned home. Following their tenure, fellows and governments were required to respond to questionnaires to determine how the fellowships were being utilized [16]. Fellowships were set up for a variety of ends, including basic clinical studies for future physicians and training of medical teachers and researchers. In 1947–48 a total of 427 fellowships were awarded to nationals of eleven countries that had suffered from wartime occupation. As conditions improved, the Organization’s work gradually moved to “broader and more balanced activities.” [17] From 1947 to 1956, 6396 fellowships were awarded to fellows from 149 countries and territories for study in 113 other countries or territories. About 29% of fellows attended courses organized or assisted by WHO, with the bulk of fellowships awarded for study in established academic centers. Most of the fellows already worked in or were destined for national and local health services, but about one tenth were granted to academic personnel in university faculties of medicine, schools of public health, and research institutions. A quarter of the fellows were women. The subjects most studied were health services (59%), control of communicable diseases (28%) and clinical and basic medical sciences (13%) [18].

Of 17,396 fellowships awarded by WHO from 1957 to 1966, 56% went to physicians, 16% to nurses, 5% to sanitary engineers and sanitarians and 23% to other health personnel or students [19]. Of these WHO fellows, about 60% studied within their region of origin (mainly Europe and the Americas) and 40% (mainly from the four other regions) left for other regions. The number of fellowships granted to staff of teaching institutions represented between 10 and 15% of the total [20]. The Fellowship program was not without critics. A report on medical manpower in the Western Pacific region argued that the program was too focused on research and that “teachers in the United States know far too little about conditions in underdeveloped countries. They are therefore unable to provide visitors with the sort of experience that will be relevant to the task back home.” [21] WHO regularly evaluated fellowships, defining as successful those in which fellows reported in a follow-up questionnaire that they had returned home and found employment relevant to the fellowship. According to a report prepared in 1968, 60–70% of fellowships were according to these criteria considered a success [22].

The WHO also conducted studies and surveys of needs, resources, and facilities in individual countries. In 1953, the organization began publishing the World Directory of Medical Schools, which contained tabular information on medical schools worldwide [23]. Successive editions were published until the seventh and final print edition appeared in 2000. (From 2000 to 2007, WHO published the directory electronically.) Surveys of health personnel and training facilities were carried out in 17 countries in Africa in 1961–1962. A conference held in 1963 estimated medical education needs in Latin America. A later study of health manpower needs in Colombia was among the reports considered at the International Conference on Health Manpower and Medical Education held in Maracay (Venezuela) in 1967. The related question of the “brain drain” of personnel from LMIC was also assessed, for example in a survey of the migration of Latin American health, scientific and engineering personnel [24]. A seminar on medical education held at Yaounde in 1966 brought together participants from 13 countries of the African Region. It was agreed that African students should be trained in institutions within the Region, where education could be oriented towards the realities of local health services [25].

### The second phase: 1969 to 1980

During this period, the earlier focus on increasing the number of health personnel and modernizing their training was integrated within a wider framework of national health services planning [26]. The WHO under the leadership of Director General, Halfdan Mahler, championed equity among nations, health system planning based on permanent infrastructure rather than foreign-organized disease-based programs, basic primary care rather than high-tech medicine, and greater emphasis on socio-economic determinants of health and illness. The new orientation that would be dubbed Health for All by the Year 2000 (HFA) was summarized in the emblematic Alma Ata Declaration of 1978 and shaped numerous reports and meetings of the period. The consequence of this framework was greater emphasis on adapting medical training to local and national needs. Central to this vision was responsiveness to “communities”. The Alma Ata Declaration would define “community” as “people living together in some form of social organization and cohesion. Its members share in varying degrees political, economic, social and cultural characteristics, as well as interests and aspirations, including health. Communities vary widely in size and socioeconomic profile, ranging from clusters of isolated homesteads to more organized villages, towns and city districts.” In real world discussions, “communities” was seldom defined and could mean anything from specific geographical units to the world outside medical schools and hospitals.

The perceived lack of personnel became an even more critical problem since the objectives of HFA required a greatly expanded workforce. In 1969–1971, WHO and UNICEF jointly undertook a comprehensive assessment of their assistance to education and training programmes in 9 countries. “In all the countries visited, there were serious shortages of health personnel.” [27] Career structures, national planning, and training facilities were all considered inadequate, as was the *quality* of training for all healthcare personnel that rarely related to the actual needs of populations; tropical medicine (now an increasingly hot topic in international health circles) [28] was often not a required subject where it was most needed. Much of the training was hospital-oriented with no attention to health in the community. Teaching equipment was poor, while textbooks in national languages were lacking. These inadequacies were linked to the increasingly visible issue of migration of trained healthcare personnel from LMIC to high-income countries (the “brain drain”). A study completed in 1977 showed a significant increase in the migration of physicians and nurses from the former to the latter and suggested that this trend was likely to increase [29]. Not all was negative, however. A WHO report on manpower worldwide from 1971 to 1976 noted the creation by 12 member states of manpower training units within governmental ministries of health and the establishment of several WHO inter-regional centres to train educators [30]. Traditional concerns with improving teaching institutions intensified, leavened by an increasingly critical view of medical schools. The Director-General of WHO, Halfdan Mahler, famously advised the president of one country to close the medical schools in his nation for 2 years, as they were the main sources of resistance to change. During that period, “they could discuss what they were supposed to do” [31]. Socrates Litsios has argued that Mahler’s critical view of medical schools and his insistence that “additional resources must not go for more hospitals and the training and support of more doctors, but should support peripheral health workers and community workers” led to the exclusion of medical education from the Alma Ata agenda [32].

The problematic state of medical training in many countries was widely acknowledged. The delegation of the USSR to the 24th World Health Assembly (WHA) in 1971 introduced a draft resolution on the subject of national training of health personnel, inviting the Director-General to examine a broad range of issues. The following year, a post in educational planning was established at WHO. During 1973, three expert committees addressed respectively postgraduate education and training in public health, continuing education for physicians, and the planning of medical education programmes that met the needs of their societies [33]. Recommendations elaborated in these committees were quickly endorsed by WHO regional offices [31].

Since 1969, a comprehensive, long-term program had been under way to train teachers of medicine and other health sciences [34]. Two institutions were designated as WHO collaborating centres: the Central Institute for Advanced Medical Studies in Moscow and the Center for the Study of Medical Education at the University of Illinois College of Medicine. The latter aimed to train educational leaders and specialists for the regional teacher-training centres that WHO was helping establish. The future directors of these regional centres took a 1-year course at the centre in Illinois and by 1974, regional teacher-training centres had been established in all WHO regions except Europe [35]. By the end of 1977, several national centres were training teachers in their own settings and languages. Collaborating centres in Iran, Israel, and Switzerland, participated in another WHO program for research in educational planning.

By 1974, the objectives of the program had evolved to take account of the WHO’s new systems-based approach. This meant conceiving training in terms of the personnel needs of the overall healthcare system. In keeping with this holistic view of health manpower, it was decided that a long-term aim of the program should be to encourage the gradual integration of educational technology centres, regional teacher-training centres, and other single-function educational units into multipurpose health manpower centres training all levels of health-care personnel able to work closely together. The goal was to encourage the training of auxiliary personnel being neglected by most countries due to opposition from medical professions and politicians [36]. The new orientation also affected the WHO’s fellowship program; one of its objectives by the early 1980s was to keep more personnel within their region by increasing training capabilities in fellows’ country of origin and, if that was not possible, within their region [37].

Another major change was the direct consideration of the role of universities in advancing HFA. Reports discussed at the 37th WHA in 1984 were presented as part of “an ongoing series of attempts to focus on the tremendous potential that universities have for influencing the outcome of health care in different settings around the world.” [38] A key aspect of HFA was community participation, which required informed communities. Hence a resolution was introduced urging Member States to encourage universities to include the concepts of HFA in the training of all categories of students and post-graduates. Furthermore HFA required action on the social, economic, and political aspects of health, making the perspectives of non-medical disciplines indispensable. Universities were invited “to conduct biomedical, epidemiological, technological, social, economic and behavioural research” but the central goal was to train healthcare workers attuned to “the health needs of the people they are to serve.” [39] This, it was recognized, would not be easy. One report suggested that an obstacle to university participation in HFA was the fear of academics that “community-oriented teaching and research will lower standards” [40]. Less charitably, it was thought that medical schools are often “trapped by the conservatism and inward, clinically-oriented guild complex” [41].

Despite the WHO’s dominant position in this field, it was not the only organization seeking to improve medical education. In 1978, the Rockefeller Foundation introduced the International Clinical Epidemiology Network (INCLEN) which began functioning 2 years later under the leadership of American health services research pioneer, Kerr White. INCLEN created programs at medical schools in developed and eventually in developing countries to train physicians in clinical epidemiology, with the aim of providing them with population-based perspectives necessary to manage limited resources [42]. In the mid-1980s, health economics and social sciences were added to the core training program, not very successfully. Unlike the WHO, INCLEN did not try to reframe the entire medical curriculum.

In 1979, “at the instigation” of the WHO, 19 medical schools formed the Network of Community-Oriented Educational Institutions for the Health Sciences. The goal was for institutions to help each other develop training programs that spoke to the health problems of local populations, notably underserved populations, outside the hospital setting. The need to respond to local needs and problems suggested that curricula be problem-based, as was occurring in newer institutions like McMaster University (Canada), Ben Gurion University (Israel), Gezirah University (Sudan) and University of lllorin (Nigeria), all among the founding institutions of the network. In addition to “community-oriented” and “problem based”, terms that were interpreted in assorted ways, there was emphasis on newer “active” pedagogical methods rather than passive rote learning; this included self directed independent study and lifelong learning. By 1990 the network had expanded to 54 member institutions and 80 associated members [43]. The largest groups were in the Americas (45) and Europe (39) with 20–22 institutions in each of the four other regions. A review of published studies on the effects of problem-based and student-centered programs found that their graduates chose careers in primary care in greater numbers than those in conventional programs who did slightly better on traditional measures of academic achievement (examinations). Attempts to measure actual clinical competence were inconclusive [44].

### The era of reports and meetings: 1980s and 1990s

The 1950s and 1960s had been characterized by efforts to export to LMIC what was then considered the best of western medical training. The 1970s and early 1980s reflected the ideals of Alma Ata, pushing schools to be responsive to perceived local and national needs. In the late 1980s, WHO entered another phase by intensifying its focus on physician training. This corresponded with the end of Mahler’s term as Director General and the decade-long tenure of his replacement, Hiroshi Nakajima. It also corresponded with the intensified work of an allied organization, the World Federation for Medical Education (WFME) – established in 1972 with the collaboration of the WHO and the World Medical Association (WMA) – seeking to introduce international standardization to medical training. The justification for this effort was the multiplication of new medical schools worldwide, many private or for profit and of unknown quality, that appeared to problematize the international mobility of medical students and physicians. There was from the beginning unacknowledged tension between the twin goals of getting medical schools to reform themselves in whatever way that they could and setting up regulatory mechanisms to control the movement of physicians internationally [45]. The WFME was led by Europeans during this period and its activities may well have originated in ongoing efforts to harmonize medical training in the European Union (EU) and to facilitate the free movement of professionals. (From 1983 to 1996, during the Presidency of Henry Walton, the WFME Central Office was located in Edinburgh; from 1996 to 2008 under the Presidency of Hans Karle, it was located in Copenhagen where it remains.) Attaining these goals became even more urgent after the fall of the Soviet Union and the subsequent expansion of the EU.

The WFME – organized in a regional structure parallel to that of the WHO – sought to directly influence national medical associations and educational facilities while the WHO urged commitment from member states to act on the recommendations produced by this collaboration. If the WFME focused on the details of medical training for physicians, such training was for the WHO only one part of a wider strategy to strengthen and scale up the entire healthcare workforce, including efforts to keep physicians and students in LMIC from emigrating to wealthier nations. Part of that strategy was to advocate relentlessly to make training reform an essential component of international and global health. Two global conferences on medical education, held in Edinburgh in 1988 and 1993, publicized and legitimized the education reform agenda of the WHO – WFME collaboration. The 1988 conference was the culmination of six regional and two national consultations conducted over the preceding 2 years [46]. This broad consultation meant that the resulting Edinburgh Declaration received the approval of medical education experts from across the world. It provided a roadmap for education reform, made up of 12 actions to guide national and institutional leaders. These actions addressed many of the same issues WHO had addressed in earlier decades and built on the concept of community-oriented medical education, promoted by the WHO since the 1970s. This meant that the education of health personnel should be relevant to the needs of the population being served and carried out in those environments where most healthcare took place, i.e. in community health facilities [47]. In addition to teaching biomedical science, medical curricula needed to include training in health promotion, take account of local resources, and instill values of social responsibility in practitioners.

The Edinburgh Declaration was approved by the 42nd WHA in 1989 [48]. Two years later, the WHO Division of Development of Human Resources for Health published an action agenda outlining a framework to guide member states in setting education standards, developing assessment tools, and monitoring reform progress. This agenda could be liberally interpreted by member states but it linked medical education reform with HFA goals, proposing that “change in medical education is a universally accepted prerequisite to the improvement of equity and equality in health care.” [49] The 1993 World Summit on Medical Education, assessed the impact of the 1988 Declaration and made further recommendations to support implementation. Although the Declaration had brought attention to medical education reform, it was conceded that the recommendations had been implemented in “many but by no means the majority of medical schools” [50]. Among the Summit’s 22 recommendations were streamlining interactions between medical education and national health systems, and promoting curriculum change, with the introduction of medical ethics, communication skills, and population based medical training particularly emphasised [51].

In 1995, the 48th WHA adopted a resolution urging member states to continue supporting collaborative efforts to define “the desired profile of the future doctor” [52]. The training requirements for this “future doctor” were outlined in the WHO’s 1996 *Global strategy for reforming medical training*. The influence of PHC and HFA were clearly visible in the strategy’s list of five key skill sets for physicians: care provider, decision-maker, communicator, community leader and manager. Biomedical and scientific knowledge was deemphasized in favor of communication and leadership skills that supported a health promotion and community- oriented approaches to medical practice [53]. Initial discussions about implementing the global strategy took place at six regional conferences, co-sponsored by the six regional associations of the WFME and the corresponding regional offices of WHO. The latter was tasked with offering technical support to member states as they implemented recommendations and monitored outcomes [54].

### The era of global standards and accreditation

In 1998, the WFME Executive Council declared its intention to expand the accreditation of medical schools worldwide [55]. International task forces produced global standards for all three levels of medical training – basic (undergraduate) medical education (BME), postgraduate (PME) and continuing professional development (CPD). The trilogy of standards was launched at the WFME World Conference in Copenhagen in 2003, held in cooperation with WHO, UNESCO and the WMA. As the first to be completed – and the main focus of education reform in many countries – the BME standards received the lionshare of commentary and assessment. They were adopted by the WFME Executive Council in June 2001 [56], and by the official launch had been tested and validated in pilot studies [57], translated into more than 12 languages, and adopted by several medical schools [58]. The WFME emphasized that the standards were not intended to weaken national authority over the education of health personnel or to make medical education uniform across the globe [59]. Rather they were intended as a tool for education reform, a reason for medical schools to undertake self-assessment exercises and identify areas for improvement.

The WHO meanwhile maintained its traditional framework of viewing physician education as one element in the larger strategy now referred to as Human Resources for Health (HRH), designed to remedy the chronic shortage of healthcare workers at all levels and improve their quality. This perspective was attracting new institutional actors, in part because it appeared that personnel shortages were threatening the attainment of the health-related Millenium Development Goals (MDGs) that had been signed in 2000 by all member states of the United Nations. The Rockefeller Foundation and several other agencies and foundations, including the Bill and Melinda Gates Foundation, funded the Joint Learning Initiative on Human Resources for Health, composed of a group of experts who produced a report in 2004 that emphasized the word “crisis”, a term that was used repeatedly to describe the healthcare personnel situation [60]. This led to a a number of international forums on HRH and two WHA resolutions in 2004 and 2005 on the International migration of health personnel [61]. Among other directives, the Director General was asked to elaborate a voluntary code of practice for the ethical international recruitment of health personnel in order to mitigate negative impacts on LMIC [62]. (This code was eventually adopted at the 63rd WHA in 2010). The World Health Report of 2006 identified 57 countries with critical shortages of health workers. The WHA of that year passed resolution WHA59.23, recommending that member states support a rapid scaling up of health worker training [63]. That same year, the WHO set up a new partnership under its auspices, The Global Health Workforce Alliance, to organize consolidated action on HRH for the next decade [64].

In the years that followed, the issue of HRH became more pressing as a consensus emerged on the need for some form of universal healthcare. In 2010, the *Lancet* published the report of the Global Independent Commission on the Education of Health Professionals for the twenty-first Century. This argued that training institutions must instill a sense of social accountability and public responsibility in students. This was, according to journal editor, Richard Horton, “nothing less than a remoralisation of health professionals’ education.” [65] The commission advocated for a curriculum based on core competencies and assessed by accreditation standards that enshrined social accountability [66]. Medical schools from across the world expressed support for these principles by participating in the process that produced the Global Consensus on Social Accountability of Medical Schools [67]. The role of social accountability in medical education was expanded in the 2012 revision of the BME standards, followed by revisions to the PME and CPD standards in 2015 [68].

Global standards were accompanied by pressure to develop accreditation programmes for medical schools as tools for quality improvement. In January 2004, a WHO-WFME strategic partnership was formed for this purpose and a joint task force was established. Consultation with member states resulted in the publication of WHO/WFME Guidelines for Accreditation [69]. These described the essential elements of accreditation: institutional self-evaluation; evaluation by the external accrediting agency; and public dissemination of the agency’s final report as well as the outcome of accreditation [70]. The guidelines provided a framework for accreditation rather than stipulating the exact content of accreditation standards, which depended on national context.

The promotion of accreditation became a central component in WHO/WFME efforts to reform medical training. One report observed that although accreditation was voluntary, incentives as well as professional and market pressures would over time make it “virtually compulsory” [71]. The addition of accreditation data to medical school directories was one such incentive. The WHO-WFME taskforce proposed the expansion of the existing WHO Directory of Medical Schools into a global database of health education and training institutions [72]. An agreement in 2007 transferred the existing WHO database to the University of Copenhagen where WFME was headquartered. The new database for medicine, called *The Avicenna Directory of Medical Schools*, was the first directory of medical schools to be based on an extensive questionnaire tested in a pilot study [73]. In 2014, the Avicenna Directory was merged with the *International Medical Education Directory* to form the *World Directory of Medical Schools* [74].

The accreditation effort was given a major boost in 2010 when the Educational Commission for Foreign Medical Graduates (ECFMG), responsible for certifying foreign graduates to attend post-graduate programs in the US and Canada, announced that starting in 2023 only graduates of medical schools that had been accredited by an approved agency would be eligible for its examinations and certification. Agencies recognized by the WFME were among those acceptable to the ECFMG [75]. The rationale once again was the perceived need to deal with quality issues posed by the rapid multiplication of medical schools, especially private schools, worldwide, and whose graduates were moving around the world. (The number of schools rose from 1600 at the turn of the millennium fo 2188 in 2010.) Increasing numbers of Americans and Canadians were undertaking medical training abroad and seeking to return home. It was the emergence of these “multinational medical schools” and of a global healthcare market that was thought to require a strong regulatory response [76]. The goal of ECFMG was nothing less than to “improve the quality, consistency and transparency of undergraduate medical education worldwide.” [77] Thus, unlike WFME, which framed accreditation as an instrument for institutional self-improvement, ECFMG emphasized the regulatory functions of accreditation. It thus shifted the focus upstream, from judging the quality of individual international medical graduates to evaluating the quality of medical schools and, even higher upstream, the quality of accreditation authorities. By 2018, the WFME recognized 14 accreditation authorities, including in Sudan, Thailand and Turkey. Another 7 accreditation authorities had formally begun applications for WFME recognition. This left about 100 accreditation authorities without this recognition. About one-third of the 183 independent nation states lacked accreditation authorities altogether [78]. As of April 2020, The WFME web site lists 21 recognized accreditation agencies, with another 13 agencies in the process of applying for recognition [79]. In June 2021, the ECFMG announced a year's delay in implementing accreditation requirements due to COVID-19.

## Discussion

Before the emergence of the WHO-WFME alliance, comment on medical training reform remained sparse and under the public radar. But as human resources and accreditation became increasingly central to GH, discussion intensified. One common theme was the difficulty of implementing effective educational reform [80]. But criticsm of the standardization strategy also emerged. A traditional reproach, going back to WHO’s early fellowships program, was that it was not relevant to the needs of LMIC, or worse, that it imposed the training standards of rich nations on poorer ones [22]. The determination of Mahler and his successors at the WHO to adapt training to local and national needs somewhat deflected this charge, except that their insistence on determining those needs (primary health care carried out largely by lower level community workers) can be interpreted as yet another example of western hubris. They simply dismissed opposition from local health personnel who, especially in colonial Africa, had been largely relegated to lower level auxiliary positions and frequently saw emphasis on auxiliary personnel as both inferior and discriminatory [81]. Such criticism persisted despite repeated assurances that training was a local matter involving local adaptation of global standards. A letter to the editor of the *Lancet* in 2014 from members of the Consortium of New Sub-Saharan African Medical Schools, which sought to develop appropriate accreditation principles for the region, warned against copying accreditation systems from developed countries in an effort to “conform to Western educational imperatives.” [82] Such admonitions have been equally directed at attempts to develop global curricula for specialties around measurable competencies [83].

As the standardization movement advanced with considerable support from medical academics [84], discussion and debate became more frequent. In some cases it was argued that standards were too expensive to implement for many countries. Richard Hays wrote in 2014 that the revised BME standards “appear to raise the bar” for medical schools, which could make them difficult to achieve for institutions in LMIC [85], It was also feared that wealthy countries like the US which are dependent on foreign graduates for their healthcare needs (about one-quarter of the US physician workforce is made up of international medical graduates) would face serious personnel shortages if such criteria are applied [78]. (One response to this fear was that the number of successful applicants, far outnumbered availale residencies so that the new standards would not significantly impact US personnel needs.) [86] Others suggested that many of these standards that followed that latest trends in Western medical education were steeped in a particular set of cultural attitudes incompatible with cultural values in many nations. Taking up a familar trope, to the effect that GH was rife with the values of colonialism [87], some critics identified the “enterprise of globalizing the medical curriculum” with a new wave of neocolonialism. Bleakley in fact has made a strong case that the language of standardization is self-contradictory, insisting on the need for local differences and diversity while promoting western standards expressed as core competencies [88]. A group of Dutch scholars refer to similar arguments about the dominant discourses around Problem-based Learning (PBL) a central element in accreditation standards, that “perpetuate neo-colonialist views and practices in health professions education globally,“ [89] only to dispute this by arguing that interpretations of PBL differ from place to place and thus do not represent a single hegemonic discourse. A more radical critical perspective argues that “Approaches imported and imposed through colonization have been accepted as preferred modern practices in the global South.” This translates into “epistemicide” in which indigenous knowledge and approaches including dreams, vision and divination have been devalued and disowned [90].

While charges of colonialism tend to conflate its very real historical sequelae with virtually all forms of unequal power, influence, and benefits, they have clearly touched a nerve. Spokesmen for WFME like Hans Karle et al. have responded that the consultative process used to develop the standards was “truly international – not Western … the core values that emerged … are likely to genuinely represent global values.” [91] A more recent response to concerns about “western bias” is that “communication with experts from all over the world stresses the view that such a trend is moderate, acceptable and unavoidable.” [92] It has also been noted that standards are process- rather than outcome-oriented and leave significant room for community, national and regional adaption. This attempt to balance global standards and local variation does not seem very convincing in regions where medical schools share an academic culture that prioritizes excellence as defined by medical schools in the Global North, to which aspiring physicians look for specialized training if not possible migration. At the very least, there has clearly been tension between forces for globalized standards and those for local variation [93]. Some writers have responded with studies showing that standards were applied with significant revisions in countries like Taiwan, South Korea and Japan, indicating that they are by design not hegemonic [94]. One wonders however how much local priorities have been compromised in the process, especially in low-income countries. At the very least, it would seem as if international safety is protected by core standards that are universal while local variations are concessions to poverty or cultural difference.

As accreditation has spread, some researchers have pointed out that there is little evidence that it actually improves the quality of medical education [95]. A 2019 scoping review of the impact of accreditation concluded that there was insufficient research to permit evaluation of its effects [96]. Others have stressed that given the expense associated with accreditation, research to determine its impact on training should be prioritised [97]. A recent review of the literature on the implementation of accreditation concluded that the WFME standards were useful, but that more systematic analysis of their use and consequences was required [98]. Part of the problem has to do with what to measure. There is some data that students from accredited schools do better on the ECFMG examinations; however there is little evidence that such exams are accurate predictors of physician quality. An alternative metric that has been proposed centers on the institutional efforts at self-improvement that accreditation promotes [99]. Here the assumption is that all such efforts are good. Given the two existing objects of evaluation, it is hard to see how credible evidence of training outcomes can be produced. There are of course new intruments being developed. The Conceptualization-Production-Usability model is made up of a “sequence of parameters” exploring school commitments in “planning actions, in implementing those actions and in ensuring actions produced the anticipated effects on society.” [100] But these are not yet proven or in common use. The possibility of widespread standardization elicits its own concerns – that better educated physicians will more easily migrate to wealthy nations while patients from wealthy nations travel to poorer ones where costs are lower, leaving few healthcare resources for local populations [101].

Other criticisms do not take issue with accreditation itself but with certain characteristics associated with it. For instance, it has been argued that the pedagogical fashions of the day, whether problem-based or competency-based curricula, are not appropriate for certain countries [102]. Others wonder about the overwhelming emphasis on medical education more generally. In an important piece, Barbara McPake et al. point out how much human resources depend on global labor markets and suggests that the conditions of these markets also requires major intervention and regulation [103]. This fits with the WHO's traditional workforce policy. Its Global Workforce Strategy of 2016 includes what it calls a “comprehensive health labour market framework for universal health coverage” [104]. It is not however clear how this is to be implemented.

## Conclusions

Standardization and accreditation are not the only forms of globalization that are transforming medical training. Some medical schools in Eastern Europe, the Caribbean and Asia (often private) now brand themselves as global schools that train students from around the world to practice medicine anywhere. Partnerships between medical schools in the Global South and those in the Global North have become common. Specialties have established graduate programs rigorously defined by the “competencies” that residents are expected to acquire [105]. But the WHO/WFME campaign for standardization and accreditation demonstrates in a uniquely focused way the history, tensions and contradictory imperatives of globalization. The move to improve medical training in LMIC has gone through several stages, from providing courses and fellowships to teach “western” medical science, to adapting training to local needs, to the current move toward merging global standards with local needs. The WHO’s *Global strategy on human resources for health* included the realisation of accreditation mechanisms for health training institutions in all countries by 2020 as a key milestone [106]. This milestone was not achieved indicating just how difficult it is to implement. Indeed across the 34 states the World Bank considers low-income, only 7 had national accrediting authorities by the end of 2018.

The fact that accreditation has yet to demonstrate that it improves actual medical performance – as opposed to examination success or self-improvement exercises – is largely irrelevant to its spread. Even those who demand better evidence of efficacy rarely reject accreditation out of hand. Some of this reflects practical realities. In an effort to improve quality and reduce risk, key western nations like the USA and Canada that certify foreign graduates seeking post-graduate study are making institutional accreditation mandatory [107]. For many countries, the supply of physicians with postgraduate training is thus dependent on implementing some form of accreditation. Aside from these practical constraints, many medical academics in LMIC who have frequently taken part of their training in Europe or North America share the culture of safety and excellence that now animates medical education in wealthy nation. Furthermore, it is hard to oppose accreditation when it is articulated as a guarantee of safety or occasion for institutional self-improvement; who can oppose such well-meaning and seemingly self-evident goals, even without convincing proof that they work. On a deeper level, accreditation, even locally modified, is part of the “audit culture” [108] that has become central to health systems everywhere and that is based on the notion that there are “correct” or best ways to provide healthcare, and that practitioners and training institutions must be “accountable” through some form of metric. Whether it is about ending practice variation through guidelines, measures for lifelong learning and periodic recertification for physicians, or new courses in professionalism and bioethics, the goal is to transform medical practice [109]. The current COVID-19 pandemic has undoubtedly disrupted the accreditation process, as it has disrupted all aspects of life, and it is not hard to imagine that its long-term persistence will substantially slow it down. But short of that, the large consensus around audit culture, safety, and some level of standardized quality make it unlikely that the push for international training standards and accreditation will abate any time soon.

## Data Availability

All materials are available online, primarily in the WHO-IRIS collection.
